# Differences of connectivity between ESRD patients with PD and HD

**DOI:** 10.1002/brb3.1708

**Published:** 2020-06-24

**Authors:** Bong Soo Park, Myungjun Seong, Junghae Ko, Si Hyung Park, Yang Wook Kim, Il Hwan Kim, Jin Han Park, Yoo Jin Lee, Seongho Park, Kang Min Park

**Affiliations:** ^1^ Department of Internal Medicine Haeundae Paik Hospital Inje University College of Medicine Busan Korea; ^2^ Department of Neurology Haeundae Paik Hospital Inje University College of Medicine Busan Korea

**Keywords:** connectivity, end‐stage renal disease, renal replacement therapy

## Abstract

**Objectives:**

The aim of this study was to investigate alterations in structural and functional brain connectivity between patients with end‐stage renal disease (ESRD) who were undergoing peritoneal dialysis (PD) and hemodialysis (HD).

**Methods:**

We enrolled 40 patients with ESRD who were undergoing PD (20 patients) and HD (20 patients). We also enrolled healthy participants as a control group. All of the subjects underwent diffusion tensor imaging (DTI) and resting‐state functional magnetic resonance imaging (rs‐fMRI). Using data from the structural and functional connectivity matrix based on DTI and rs‐fMRI, we calculated several network measures using graph theoretical analysis.

**Results:**

The measures of global structural connectivity were significantly different between the patients with ESRD who were undergoing PD and healthy subjects. The global efficiency and local efficiency in the patients with PD were significantly decreased compared with those in healthy participants. However, all of the measures of global structural connectivity in the patients with HD were not different from those in healthy participants. Conversely, in the global functional connectivity, the characteristic path length was significantly increased and the small‐worldness index was decreased in patients with HD. However, the measures of the global functional connectivity in the patients with PD were not different from those in healthy subjects.

**Conclusion:**

This study revealed that alterations in structural and functional connectivity in patients who were undergoing PD and HD were different than those in healthy controls. These findings suggest that brain networks may be affected by different types of renal replacement therapy.

## Graphical abstract

Alterations in structural and functional connectivity in ESRD patients who were undergoing PD and HD were different than those in healthy controls. The measures of global structural connectivity were significantly different between the patients with ESRD who were undergoing PD and healthy subjects. In the global functional connectivity, the network measures in patients with HD were different from those of the healthy controls.

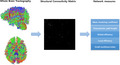

## 1 INTRODUCTION

End‐stage renal disease (ESRD) is defined by an advanced stage of chronic kidney disease with a glomerular filtration rate (GFR) <15 ml/min/1.73 m^2^. Patients with ESRD may often suffer from various complications characterized by multi‐organ dysfunction. Of these complications, neurological problems, such as peripheral neuropathy, uremic encephalopathy or cognitive impairment, have been widely recognized (Jabbari & Vaziri, 2018; Kallenberg et al., 2016; Murray et al., 2006). Renal replacement therapies, including kidney transplantation, peritoneal dialysis (PD), and hemodialysis (HD), are commonly used for the treatment of patients with ESRD. Although kidney transplantation is generally the optimal method among these therapies in terms of survival rates and quality of life, most patients remain on PD or HD because of the shortage of donor kidneys and specific situations of patients who have multiple medical complications or malignancies (Fenton et al., 1997).

The optimal choice of dialysis modality remains a matter of debate, and numerous observational studies have been conducted to investigate which dialysis modality is associated with better survival rates of patients with ESRD; however, a clear consensus has not been reached (McDonald, Marshall, Johnson, & Polkinghorne, 2009; Yeates et al., 2012). Furthermore, PD and HD both have advantages and disadvantages, but the selection of the modality can be determined by the patient's preference, age, or other co‐morbidities. HD removes waste products in the blood, such as urea, creatinine, or potassium, using a mechanical method that is more effective and efficient than PD (high dialysis efficiency). HD is also considered to be more convenient than PD (less patient responsibility). However, HD vascular access is associated with numerous complications, such as infection, stenosis, or occlusion. Unlike PD, which is continuously performed, HD can lead to electrolyte anomalies and accumulation of water during the inter‐dialytic periods. Although substance exchange is inefficient during PD, this modality is also associated with several benefits, such as preserving residual renal function. Additionally, this procedure does not require patients to visit the hospital; therefore, PD is also associated with high patient satisfaction. However, disadvantages of PD include weight gain, hyperglycemia caused by glucose‐containing PD dialyzate, peritonitis, and technical failure with membrane failure. Although both PD and HD are widely used, HD is more commonly used than PD because of convenience. Overall, about 90% of patients with ESRD in many countries, including Korea, are on HD (available at http://www.ksn.or.kr; Sinnakirouchenan & Holley, 2011). However, the results of prospective observational studies and large‐scale national registry showed no significant difference in survival rates between patients who underwent PD and HD (Sanabria et al., 2008; Weinhandl et al., 2010).

There have been several studies on brain morphology and connectivity in patients with ESRD using variable MR sequences, such as diffusion tensor imaging (DTI), voxel‐based volumetry based on T1‐weighted images, magnetic resonance spectroscopy, and arterial spin‐labeling MR perfusion imaging.(Dong et al., 2018; Kim et al., 2011; Li et al., 2016; Prohovnik et al., 2007; Qiu et al., 2014; Tryc et al., 2011; Zhang et al., 2013) Studies on quantitative brain analysis have revealed that the total cortical and subcortical volume is reduced in patients with ESRD, and this is associated with cognitive impairment (Zhang et al., 2013). They have also demonstrated that the cortical thickness of patients with ESRD is decreased as compared with that of healthy controls, especially in the frontal cortex (Dong et al., 2018). Findings from these studies may indicate that cognitive impairment in patients with ESRD is associated with these structural abnormalities in the brain. Furthermore, the structural and functional connectivity of the brain in patients with ESRD have been analyzed. A study using graph theoretical analysis based on resting‐state functional magnetic resonance imaging (rs‐fMRI) demonstrated that abnormal intrinsic connectivity pattern of whole‐brain functional networks occurs in patients with ESRD. Additionally, patients with ESRD have decreased functional connectivity in the left inferior parietal and precuneus within the brain network and increased connectivity in depression‐related regions, including the bilateral inferior frontal gyrus and the right superior temporal gyrus (Li et al., 2016). A recently published study reported that cognitive function is more preserved in patients with ESRD undergoing PD than that in patients undergoing HD; however, this has only been evaluated by two validated neurocognitive tests, not by brain imaging studies. Further, these comparison studies are rare, and most of the previous studies that have investigated brain connectivity have only enrolled patients with ESRD who were undergoing HD (Neumann, Mau, Wienke, & Girndt, 2018). Additionally, there have been no studies that have directly compared differences in brain connectivity between patients with ESRD who were undergoing PD and HD.

The aim of this study was to investigate alterations of structural and functional brain connectivity between patients with ESRD who were undergoing PD and HD and healthy controls using graph theoretical analysis based on DTI and rs‐fMRI. In addition, we analyzed differences in cognitive impairment between patients with ESRD who were undergoing PD and those who were undergoing HD.

## 2 MATERIALS AND METHOD

### 2.1 Subjects

This study was conducted with the approval of our institution's review board, and written informed consent was obtained from all participants. This study was prospectively performed in a single tertiary hospital. We enrolled 40 patients with neurologically asymptomatic ESRD who were undergoing PD (20 patients) and HD (20 patients) from October 2018 to March 2019. Patients were eligible for participation if they had a GFR < 15 ml/min/1.73 m^2^ and required renal replacement therapy, were undergoing dialysis for more than 3 months, and had no previous history of neurological or psychiatric disorders (Tryc et al., 2011).

We also enrolled an age‐ and sex‐matched control group of 40 healthy participants without significant medical, neurological, or psychiatric histories. The patients with ESRD and healthy controls had normal brain MRI findings upon visual inspection.

### 2.2 Brain MRI

All subjects in this study underwent MRI using the same imaging protocol. All scans were performed using a 3.0 T MRI scanner equipped with a 32‐channel head coil (AchievaTx, Phillips Healthcare, Best, The Netherlands). We obtained sagittal‐oriented three‐dimensional T2‐ and T1‐weighted images (TI = 1,300 ms, TR/TE = 8.6/3.96 ms, Flip angle (FA) = 8°, and a 1 mm^3^ isotropic voxel size) and coronal‐oriented three‐dimensional fluid‐attenuated inversion recovery images in order to evaluate structural lesions on their brains. Moreover, all subjects underwent DTI and rs‐fMRI that were suitable for graph theoretical analysis. DTI was performed using spin‐echo single‐shot echo‐planar pulse sequences with a total of 32 different diffusion directions (TR/TE = 8620/85 ms, FA = 90°, slice thickness = 2.25 mm, acquisition matrix = 120 × 120, FOV = 240 × 240 mm^2^, and *b*‐value = 1,000 s/mm^2^). The rs‐fMRI was performed using multi‐slice echo‐planar imaging (EPI) sequences (TR/TE = 3,000/30 ms, FA = 65°, slice thickness = 4.4 mm, acquisition matrix = 128 × 128, FOV = 220 × 220 mm^2^, scan time = 7 min 30 s).

### 2.3 Image processing and analysis

Voxel‐based morphometry (VBM) based on T1‐weighted images was utilized to analyze volumetric difference. Image processing was performed using the computational anatomy toolbox, implemented in the Statistical Parametric Mapping software packages (SPM, version 12, Functional Imaging Laboratories, London, UK) running under Matlab 2019B (MathWorks, Sherborn, MA, USA). The VBM data were processed in the standard manner, such as gray matter segmentation, spatial smoothing with 5 mm Gaussian kernel, and statistical analysis with two‐sample *t* tests (patients versus normal controls). Age, gender, and total intracranial volume were considered as covariates.

**FIGURE 1:**
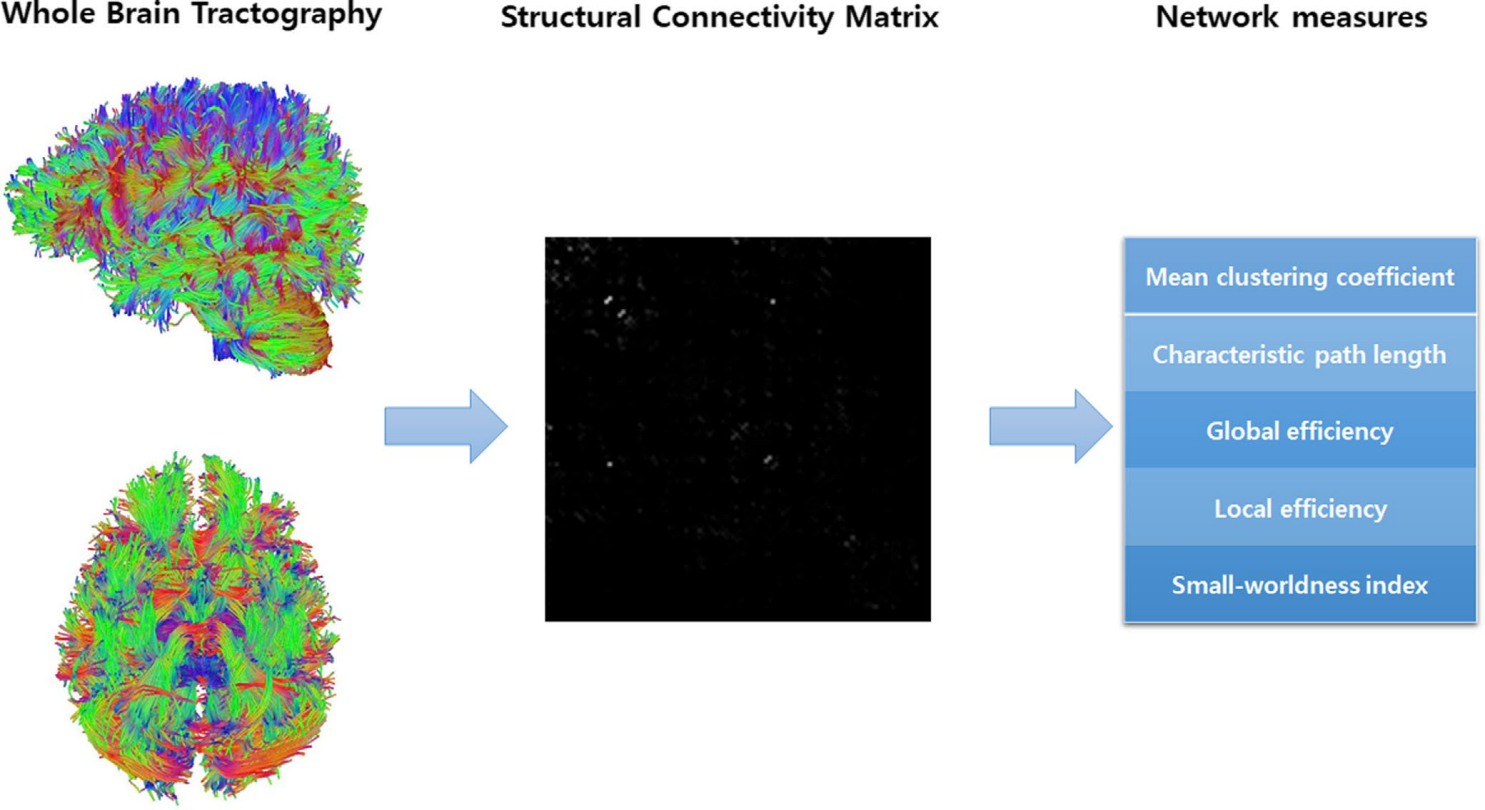
Structural connectivity. A connectivity matrix is generated from the whole brain tractography, and the graph theoretical network measures are calculated from the connectivity matrix

DTI was used to evaluate structural connectivity, and information from DTI was evaluated using the DSI studio (http://dsi‐studio.labsolver.org). The procedures for the graph theoretical analysis are described hereinafter. First, we created a tractography from the DTI data, which included reading and parsing DICOM files, reconstructing to characterize the major diffusion direction of the fiber, and fiber tracking. Next, we generated a connectivity matrix, which was calculated using the count of the connecting tracts. We used the Automated Anatomical Labeling (AAL) template for brain parcellation, and every white matter fiber was evaluated for extreme points. This step included obtaining a whole‐brain fiber track, placing seeding regions in the whole brain, establishing spatial normalization, defining the region of interest, and creating a connectivity matrix. Finally, we calculated the graph theoretical network measures from the connectivity matrix (Figure 1).

The rs‐fMRI data were analyzed using the SPM, and the functional connectivity toolbox Conn (Cognitive and Affective Neuroscience Laboratory, Massachusetts Institute of Technology, Cambridge, USA).The rs‐fMRI data were preprocessed using the standard spatial preprocessing steps of realignment, slice‐time correction, co‐registration, normalization in Montreal Neurological Institute (MNI) space, and smoothing with a 6‐mm Gaussian kernel. Functional connectivity analysis was then performed using the CONN toolbox (version 17).

Using data from the structural and functional connectivity matrix based on DTI and rs‐fMRI, we calculated the network measures, including global efficiency, local efficiency, mean clustering coefficient, characteristic path length, and small‐worldness index.

### 2.4 Neuropsychological tests

We evaluated the cognition of patients with ESRD using the Korean version of the Consortium to Establish a Registry for Alzheimer's Disease Assessment Packet (CERAD‐K) (Lee et al., 2002). This neuropsychological test consists of measures for assessing frontal/executive function, language, memory, and visuospatial functions. The following tests were scored: the Verbal Fluency test, the Trail Making Test B, Stroop test color reading, the Korean version of the Boston Naming Test (K‐BNT), 10‐word list immediate memory, 15‐min delayed recall and recognition, delayed figure recall, constructional praxis, and the Mini‐Mental State Examination (MMSE) in the CERAD‐K (MMSE‐KC). The Verbal fluency test, the Trail Making Test B test, and the Stroop test were performed to assess the frontal/executive functioning of participants; the K‐BNT was used for language function, word list immediate, and delayed recall and recognition; delayed figure recall tests were performed to evaluate memory; and constructional praxis and MMSE‐KC were completed to evaluate visuospatial function and global cognition, respectively. Cognition was considered impaired if scores were more than the 1.5 standard deviations (SDs) below the age‐ and education‐adjusted norms. Patients were considered cognitively impaired if at least two cognitive domains were impaired on the CERAD‐K. The neuropsychological tests were administered by a board‐certified clinical neuropsychologist.

### 2.5 Statistical analysis

Comparisons were analyzed using the chi‐squared test for categorical variables and the Student's *t* test or Mann‐Whitney test for numerical variables. Categorical variables were presented in terms of both frequency and percentage. Numerical variables are presented as mean ± *SD*. For all analyses, *p* < .05 was considered statistically significant. We corrected for multiple comparisons using the false discovery rate method when the local connectivity analysis was conducted. All of the statistical tests were performed using MedCalc Statistical software version 19.0.3 (MedCalc Software bvba, Ostend, Belgium).

## 3 RESULTS

### 3.1 Demographic and laboratory characteristics of subjects

The mean age of the patients with ESRD was 62 years. Of the 40 patients with ESRD, 18 patients were male, and 22 patients were female (Table 1). The demographic characteristics were not different between the ESRD patients who were undergoing PD and HD, except for the duration of dialysis. The duration of dialysis in the patients who were undergoing PD was significantly longer than that in those who were undergoing HD. Laboratory tests revealed that the serum levels of creatinine and total CO_2_ contents in the patients who were undergoing PD were significantly higher than those in the patients who were undergoing HD. However, albumin levels were lower in the patients who were undergoing PD than those in patients who were undergoing HD. Other demographic and laboratory characteristics are described in Table 1.

**TABLE 1:** Differences of demographic, laboratory, and neuropsychological data between end‐stage renal disease patients with peritoneal dialysis and hemodialysis

Variables	Patients with ESRD (*N* = 40)	Patients with PD (*N* = 20)	Patients with HD (*N* = 20)	*p*‐value^a^
Demographic data				
Age, years (*SD*)	62.9 (6.59)	60.95 (5.414)	64.85 (7.199)	.060
Men, *N* (%)	18 (45)	10 (50)	8 (40)	.525
Dialysis year, months (*SD*)	46.5 (52.53)	63.1 (66.262)	29.9 (26.338)	.049*
Education, years (*SD*)	10.4 (4.1)	10.60 (3.633)	10.15 (4.545)	.883
Dialysis adequacy, *N* (%)	36 (90)	16 (80)	20 (100)	.106
Laboratory data				
Hemoglobin, g/dl (*SD*)	10.41 (1.17)	10.41 (1.51)	10.42 (0.74)	.969
Hematocrit, % (*SD*)	31.91 (3.68)	31.88 (4.75)	31.95 (2.27)	.950
Protein, g/dl (*SD*)	6.55 (0.63)	6.39 (0.65)	6.72 (0.58)	.056
Albumin, g/dl (*SD*)	3.8 (0.34)	3.64 (0.27)	3.96 (0.34)	.003*
Aspatate aminotransferase, U/L (*SD*)	21.1 (6.89)	21.4 (6.11)	20.8 (7.75)	.787
Alanine aminotrasferase, U/L (*SD*)	20.13 (10.7)	20.2 (9.50)	20.05 (12.02)	.429
BUN, mg/dl (*SD*)	58.84 (16.72)	56.75 (14.69)	60.92 (18.68)	.142
Creatinine, mg/dl (*SD*)	9.00 (2.5)	9.97 (2.21)	8.04 (2.45)	.013*
Sodium, mmol/L (*SD*)	138.78 (3.25)	138.65 (3.89)	138.9 (2.55)	.779
Potassium, mmol/L (*SD*)	4.81 (0.65)	4.65 (0.54)	4.97 (0.72)	.081
Chloride, mmol/L (*SD*)	99.23 (4.16)	98.55 (4.32)	99.9 (3.98)	.311
Calcium, mg/dl (*SD*)	8.49 (0.68)	8.39 (0.70)	8.59 (0.66)	.375
Phosphate, mg/dl (*SD*)	4.85 (0.94)	5.05 (1.03)	4.66 (0.81)	.289
Parathyroid hormone, pg/mL (*SD*)	286.63 (209.31)	317.81 (260.93)	255.45 (140.69)	.904
Total CO_2_ contents, mmol/L (*SD*)	24.68 (2.62)	25.6 (2.46)	23.76 (2.49)	.024*
Neuropsychological data (*z*‐score)				
Frontal/Executive function				
Verbal Fluency Test (*SD*)	−0.67 (0.94)	−0.54 (1.18)	−0.80 (0.64)	.393
Trail Making B (*SD*)	−1.22 (1.11)	−1.68 (0.84)	−0.77 (1.18)	.008*
Stroop Test (reading color words) (*SD*)	−1.03 (1.10)	−0.81 (1.17)	−1.25 (1.01)	.213
Language				
Modified Boston Naming (*SD*)	0.25 (0.86)	0.53 (0.83)	−0.03 (0.81)	.036*
Verbal memory				
Word List Memory (*SD*)	−0.03 (1.08)	0.33 (0.88)	−0.38 (1.17)	.036*
Word List Recall (*SD*)	−0.16 (1.12)	0.32 (0.81)	−0.63 (1.20)	.005*
Word List Recognition (*SD*)	0.11 (0.93)	0.29 (0.56)	−0.07 (1.18)	.218
Visual memory				
Constructional Recall (*SD*)	−0.37 (0.95)	−0.47 (0.92)	−0.28 (0.99)	.539
Visuospatial function				
Constructional Praxis (*SD*)	−0.83 (1.31)	−0.99 (1.42)	−0.67 (1.20)	.437
Global cognition				
MMSE‐KC (*SD*)	0.24 (0.89)	0.12 (0.78)	0.35 (0.99)	.424
MMSE‐KC, raw score (*SD*)	26.9 (2.8)	26.8 (2.5)	27.1 (3.1)	.736

Abbreviations: ESRD, end‐stage renal disease; HD, hemodialysis; MMSE‐KC, MMSE in the CERAD‐K; PD, peritoneal dialysis; *SD*, standard deviations.

^a^ Statistical differences between patients with PD and HD.

* *p* < .05.

### 3.2 Structural volumes in ESRD patients undergoing PD and HD

**FIGURE 2:**
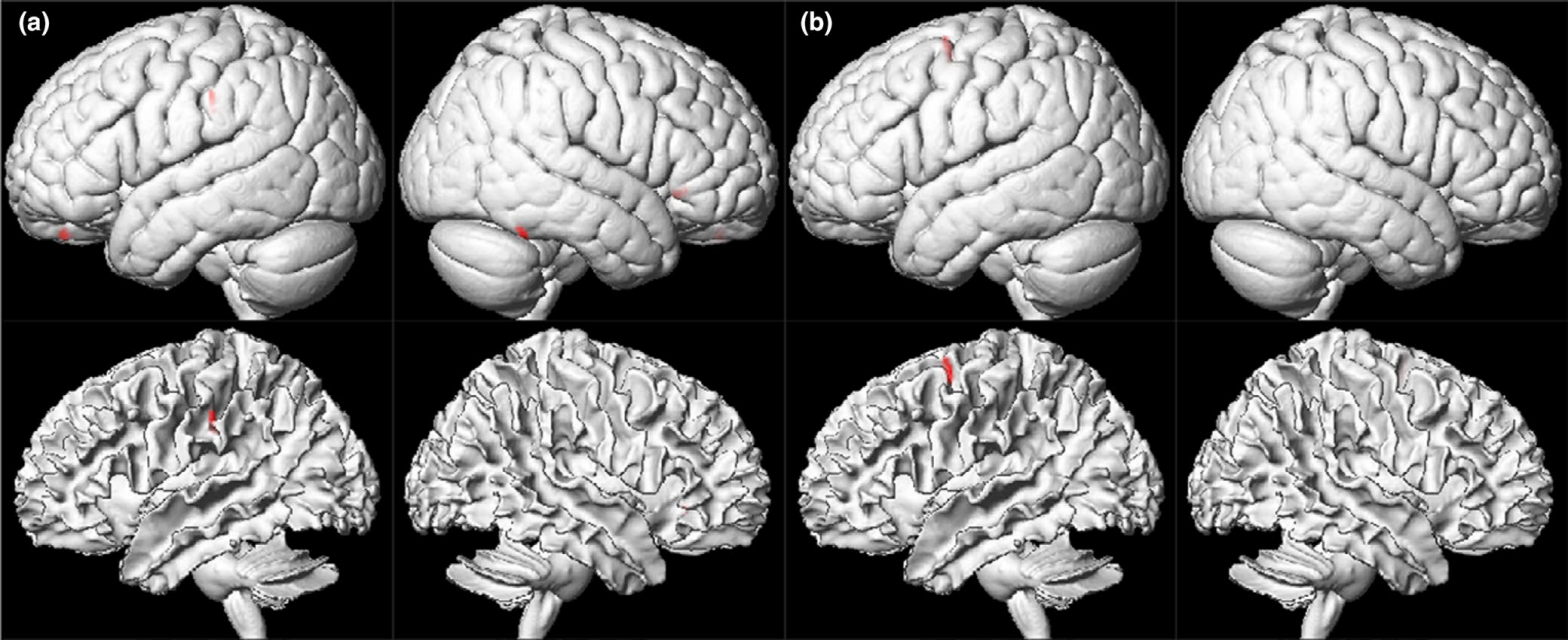
Structural volumes. The voxel‐based morphometry analysis revealed that the end‐stage renal disease patients with peritoneal dialysis exhibited significant reductions in the gray matter volumes of the right inferior temporal, left superior orbital, right inferior frontal, and left inferior parietal cortex, and the end‐stage renal patients disease patients with hemodialysis exhibited significant reductions in the gray matter volume of the left superior frontal cortex compared to healthy controls at the statistical significance level of *p* < .05 after correcting for the false discovery rate and applying an extent threshold of 30 voxels

The VBM analysis revealed that the ESRD patients with PD exhibited significant reductions in the gray matter volumes of the right inferior temporal, left superior orbital, right inferior frontal, and left inferior parietal cortex (number of voxels and peak intensity, 70 and 1.79; 36 and 2.18; 32 and 1.98; 53 and 2.07; respectively), and the ESRD patients with HD also exhibited significant reductions in the gray matter volume of the left superior frontal cortex compared to healthy controls (number of voxels and peak intensity, 61 and 2.13) (Figure 2). However, there were no structures with increased gray matter volumes in ESRD patients with PD and HD compared to the control subjects.

### 3.3 Global structural and functional connectivity in ESRD patients undergoing PD and HD

The measures of global structural connectivity were significantly different between patients with ESRD who were undergoing PD and healthy subjects. The global efficiency and local efficiency in the patients who were undergoing PD were significantly lower than those in the healthy subjects (Table 2). However, there were no differences in the measures of global structural connectivity between the patients with ESRD who were undergoing HD and healthy participants.

**TABLE 2:** Differences of the measures of global structural connectivity between end‐stage renal disease patients with peritoneal and hemodialysis compared to healthy subjects

Variable	Patients with PD			Patients with HD			Healthy controls	
	Mean	*SD*	*p*‐value^a^	Mean	*SD*	*p*‐value^b^	Mean	*SD*
Mean clustering coefficient	0.1347	0.0516	.5683	0.1317	0.0428	.6884	0.1259	.0563
Characteristic path length	3.9911	0.3284	.8508	3.9033	0.3592	.5170	3.9715	.3884
Global efficiency	0.8429	0.0876	.0002*	0.8973	0.0573	.0543	0.9377	.0820
Local efficiency	1.0594	0.1511	.0087*	1.1645	0.1490	.5694	1.1918	.1831
Small‐worldness index	0.0869	0.0350	.7621	0.0896	0.0298	.9434	0.0904	.0436

Abbreviations: HD, hemodialysis; PD, peritoneal dialysis; *SD*, standard deviations.

^a^ Statistical differences between patients with PD and healthy controls.

^b^ Statistical differences between patients with HD and healthy controls.

* *p* < .05.

In terms of functional connectivity, the characteristic path length was significantly increased and the small‐worldness index was decreased in patients who were undergoing HD as compared with those in healthy participants. However, the measures of global functional connectivity in the patients who were undergoing PD were not different from those in healthy subjects (Table 3).

**TABLE 3:** Differences of the measures of global functional connectivity between end‐stage renal disease patients with peritoneal and hemodialysis compared to healthy subjects

Variable	Patients with PD			Patients with HD			Healthy controls	
	Mean	*SD*	*p*‐value^a^	Mean	*SD*	*p*‐value^b^	Mean	*SD*
Mean clustering coefficient	0.5342	0.0335	.5503	0.5292	0.0381	.9297	0.5283	.0350
Characteristic path length	2.3340	0.1082	.1727	2.3785	0.1872	.0305*	2.2956	.0933
Global efficiency	0.4958	0.0184	.5164	0.4900	0.0243	.1042	0.4989	.0158
Local efficiency	0.7350	0.0171	.5680	0.7253	0.0260	.2986	0.7319	.0200
Small‐worldness index	0.2288	0.0090	.6552	0.2230	0.0138	.0314*	0.2300	.0098

Abbreviations: HD, hemodialysis; PD, peritoneal dialysis; *SD*, standard deviations.

^a^ Statistical differences between patients with PD and healthy controls.

^b^ Statistical differences between patients with HD and healthy controls.

* *p* < .05.

### 3.4 Local structural and functional connectivity in ESRD patients undergoing PD and HD

There were significant alterations of local structural and functional connectivity between patients with ESRD who were undergoing PD and those who were undergoing HD.

**FIGURE 3:**
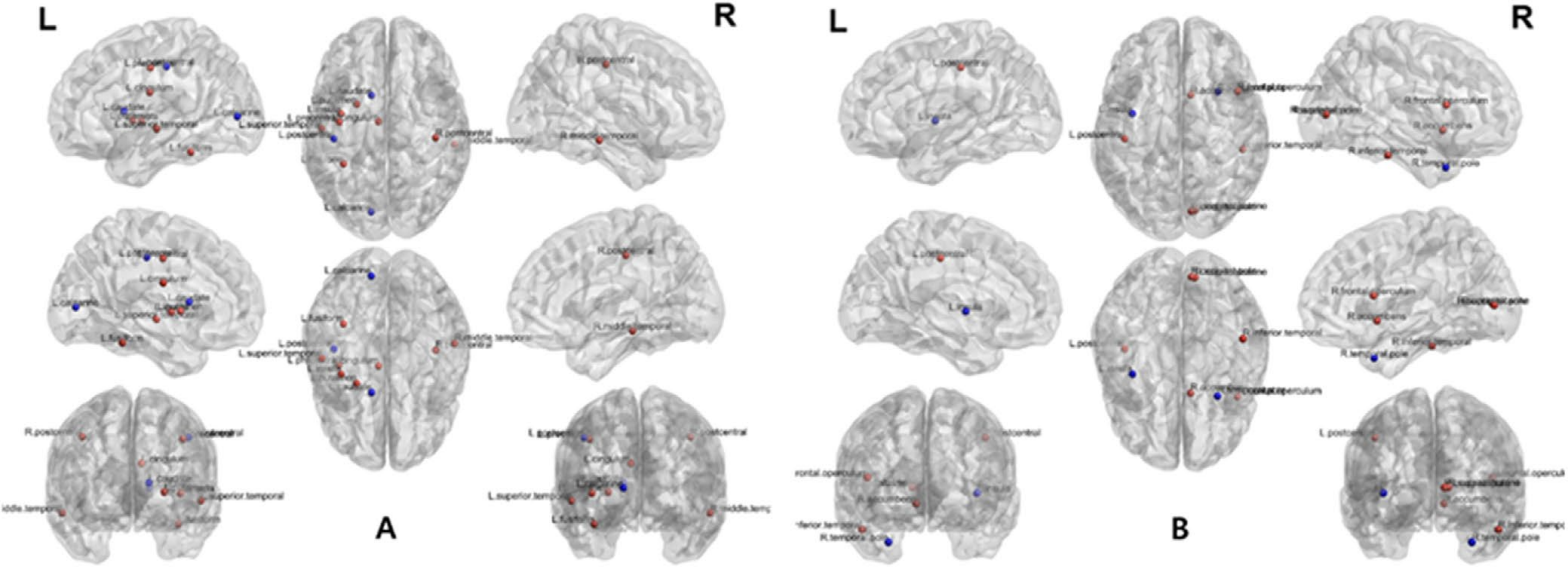
Differences of local structural and functional connectivity between end‐stage renal disease patients with peritoneal dialysis and healthy subjects (*p* < .05 with multiple corrections). Alterations in the local structural (a) and functional (b) brain connectivity in the patients undergoing peritoneal dialysis. Red circles indicate the nodes with increased betweenness centrality, whereas blue circles represent the nodes with decreased betweenness centrality in the patients undergoing peritoneal dialysis as compared with those of healthy controls

In the patients who were undergoing PD, the structural betweenness centrality of the left calcarine, left caudate, left posterior cingulum, left fusiform, left insula, right and left postcentral, left precentral, left putamen right middle temporal, and left superior temporal cortex was significantly different from that in healthy subjects (Figure 3). The functional betweenness centrality of the right accumbens, right inferior temporal, right frontal operculum, left insular cortex, right occipital pole, left postcentral, right supracalcarine, and right temporal pole cortex was significantly different from that in healthy subjects.

**FIGURE 4:**
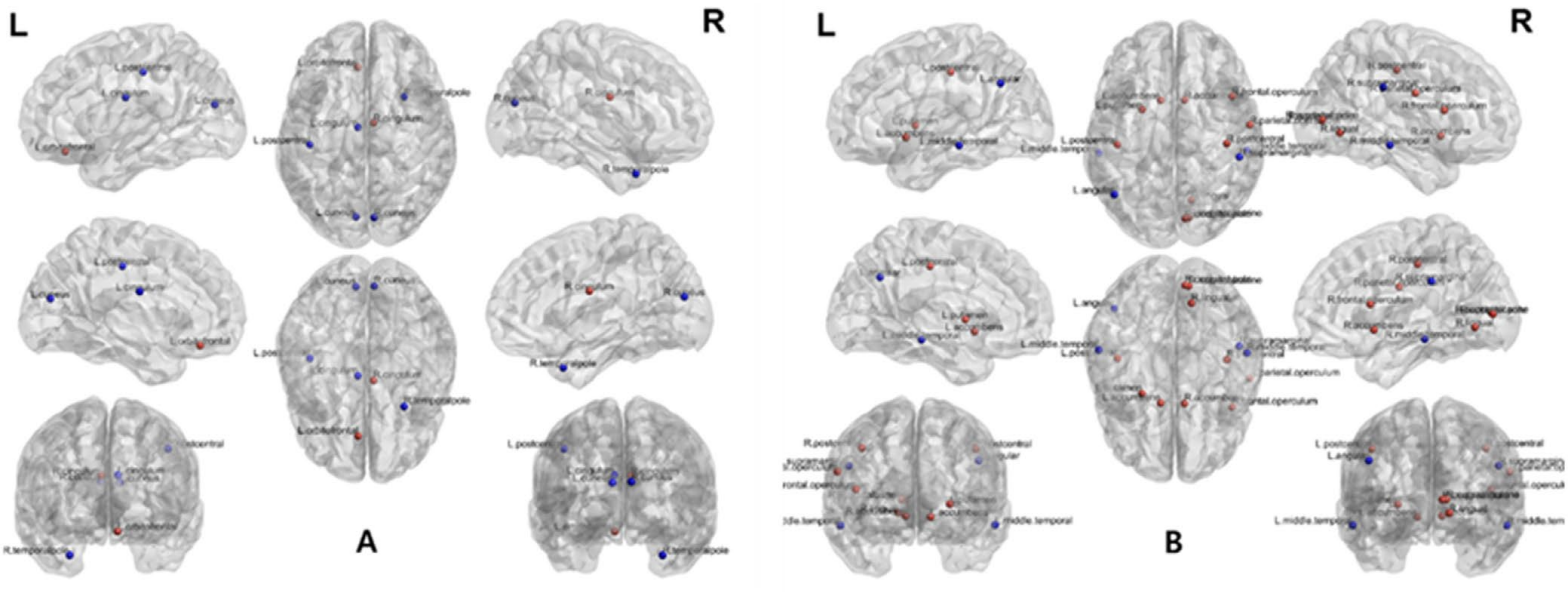
Differences of local structural and functional connectivity between end‐stage renal disease patients with hemodialysis and healthy subjects (*p* < .05 with multiple corrections). Alterations in the local structural (a) functional (b) brain connectivity in the patients undergoing hemodialysis. Red circles indicate the nodes with increased betweenness centrality, whereas blue circles represent the nodes with decreased betweenness centrality in the patients who were undergoing hemodialysis as compared with those of healthy controls

In the patients who were undergoing HD, the structural betweenness centrality of the right and left cingulum, right and left cuneus, left superior orbitofrontal cortex, left postcentral, and right superior temporal pole cortex was significantly different from that in healthy subjects (Figure 4). The functional betweenness centrality of the right and left accumbens, left angular, right supramarginal, right frontal operculum, right lingual, right occipital pole, right and left middle temporal, right parietal operculum, right and left postcentral, left putamen, right supracalcarine, and right middle temporal cortex was significantly different from that in healthy subjects.

### 3.5 Neuropsychological tests

Overall, 16 of the 20 patients (80%) who were undergoing HD had cognitive impairments (CI). Seven patients (35%) had visuospatial dysfunction, six patients (30%) had frontal/executive dysfunction, five patients (25%) had verbal memory impairment, three patients (15%) had visual memory impairment, and one patient (5%) had language dysfunction. Overall, 13 of the 20 (65%) patients who were undergoing PD had CI. Nine patients (45%) had visuospatial impairment, eight patients (40%) had frontal/executive dysfunction, two patients (10%) had visual memory dysfunction, one patient (5%) had language dysfunction, and one patient (5%) had verbal memory dysfunction.

The language and verbal memory of patients who were undergoing HD were more impaired than the language and verbal memory of patients who were undergoing PD. However, frontal/executive functioning in patients who were undergoing PD was more impaired than that of patients who were undergoing HD. There were no significant differences in the other neuropsychological test, including visual memory, visuospatial function, and global cognition, between patients who were undergoing PD and those who were undergoing HD.

## 4 DISCUSSION

We investigated alterations in structural and functional brain connectivity in patients with ESRD who were undergoing PD and HD and compared these alterations with those in healthy participants using graph theoretical analysis based on DTI and rs‐fMRI. Our findings indicated that there were significant differences in global connectivity according to renal replacement therapy. Global structural connectivity was decreased in patients who were undergoing PD as compared with that of healthy participants, and global functional connectivity was decreased in patients who were undergoing HD as compared with that of healthy controls. In addition, the regions where alterations in local connectivity were observed were different between the patients who were undergoing PD and HD and healthy participants. Furthermore, we found that more than two thirds of the patients with ESRD had CI. Most of them had visuospatial dysfunction or frontal/executive dysfunction. The language and verbal memory of patients who were undergoing HD were more impaired than those of patients who were undergoing PD; however, frontal/executive functioning in patients who were undergoing PD was more impaired than that in patients who were undergoing HD. Furthermore, we confirmed that the significant reductions of the gray matter volumes in ESRD patients with PD and HD compared to healthy controls.

In the graph theory, the global efficiency represents the efficiency of information transfer from one region to the whole network, and it is computed as the average nodal efficiency of all nodes (Hallquist & Hillary, 2019; Vecchio, Miraglia, & Maria, 2017). The local efficiency defines the efficiency of information transfer from each region to the neighboring regions, and the local efficiency of a network is conventionally defined as the average of the local efficiencies of all nodes (Hallquist & Hillary, 2019; Vecchio et al., 2017). The characteristic path length is defined as the average number of edges in the shortest path between all pairs of nodes. The small‐worldness index is related to network efficiency for brain networks and relies on the global transitivity of the network and its average shortest path length. The global efficiency and characteristic path length reflect the integration of the brain network, while local efficiency indicates the degree of segregation in the brain network (Hallquist & Hillary, 2019; Vecchio et al., 2017). Higher values of these network measures are associated with a more effective brain network. Thus, in the patients who were undergoing PD, the reduced global and local efficiency in structural connectivity analysis implied that they had an inefficient structural brain network. In the patients who were undergoing HD, decreased characteristic path length and small‐worldness index in the functional connectivity suggested that these patients had an impaired functional brain network.

Interestingly, we found that the patients who were undergoing PD had decreased global structural connectivity, whereas those with HD had decreased global functional connectivity. Brain connectivity is crucial to elucidating how neurons and neural networks process information. Structural connectivity is an anatomical concept of connectivity that refers to the existence and structural integrity of tracts that connect different brain areas, whereas functional connectivity refers to the statistical dependence of the signal from different areas and reveals the functionally integrated relationship between spatially separated brain regions. Structural and functional connectivity complementarily reflect the networks of the brain. Because alterations of structural connectivity usually cause changes in functional connectivity, these systems are intrinsically related to each other. A previous study demonstrated that resting‐state functional connectivity was variable and frequently present between regions without direct structural linkage; however, its strength, persistence, and spatial statistics were constrained by the large‐scale anatomical structure (Honey et al., 2009). Another study revealed that the functional, but not the effective connectivity matrix, closely resembled the underlying structural matrix (Honey et al., 2009). Both of these studies suggested that structural and functional connectivity share a strong relationship. However, this relationship is very complex and has not been not well defined (Davis, Kragel, Madden, & Cabeza, 2012). A study also showed that anatomical alignment of functional and structural connectivity seemed restricted to specific networks and tracts and that changes in structural and functional connectivity were not necessarily strongly correlated (Fjell et al., 2017). Previous studies also demonstrated that structural deficits were not directly related to functional abnormalities (Ikuta et al., 2014).

Functional connectivity is not always consistent with structural connectivity. Whether structural or functional connectivity is important and what causes dissociations between structural and functional connectivity is not clearly determined. Several previous studies have demonstrated that clinical presentations of many neuropsychiatric diseases, especially schizophrenia, are less correlated with structural connectivity of the brain, and more strongly correlated with functional connectivity with behavioral performance and emotional measures (Greicius, 2008; Heuvel et al., 2013). Furthermore, the study in patients with spastic diplegic cerebral palsy showed that the motor network had a significantly lower functional connectivity efficiency over the intact structural connectivity and a lower structure‐function coupling than the control group (Lee et al., 2017). All of these studies suggested that functional connectivity, rather than structural connectivity, was closely reflects clinical manifestations in the various neuropsychiatric diseases.

In this study, neuropsychological tests revealed that the incidence of CI in patients who were undergoing HD impaired functional connectivity with preserved structural connectivity was higher than that of patients who were undergoing PD, who had preserved functional connectivity with impaired structural connectivity. Furthermore, we found that more than two third of asymptomatic patients with ESRD had CI. Considering including asymptomatic patients with ESRD, these results implied that CI in patients with ESRD was underdiagnosed. A previous study using graph theoretical analysis in healthy subjects revealed a significant correlation between IQ and network measures derived from graph theoretical analysis, indicating that brain connectivity is related to cognitive function (Williamson, Altaye, & Kadis, 2019). Thus, our study suggested that the alterations of structural and functional connectivity based on brain MRI could be a potential index to reflect the CI in patients with ESRD.

This is the first study to investigate the difference of the structural and functional connectivity between patients with patients who were undergoing PD and HD. There were multiple strengths associated with our study. For example, we only enrolled patients with neurologically asymptomatic ESRD who had normal brain MRI on visual inspection in order to exclude other factors that may have affected brain connectivity. Furthermore, we simultaneously investigated structural and functional connectivity based on DTI and rs‐fMRI, respectively. In addition, we included detailed neuropsychological evaluations and analyzed the association between the network measures of brain connectivity and cognitive function. However, there were also several limitations of this study. First, the size of the dataset was small because we conducted a small‐scale, single‐center study. Second, because this was cross‐sectional study, we only observed the structural and functional connectivity and cognitive function in patients with ESRD at one‐time point. Follow‐up longitudinal studies may provide further insight into the time course of alterations in brain connectivity and cognitive function. Future studies should also compare alterations in structural and functional connectivity before and after patients with ESRD undergo renal replacement therapies, such as PD, HD, and kidney replacement. This information may help identify alterations in brain connectivity caused by the uremic state and determine the effects of renal replacement therapy on brain connectivity and neuropsychiatric symptoms. Third, some of the baseline characteristics, such as the duration of dialysis and some laboratory data, of patients who were undergoing PD and HD were different. Furthermore, several co‐morbidities of ESRD, such as anemia, hypertension, and diabetes, can influence functional connectivity based on rs‐fMRI, and we were unable exclude these effects.

## 5 CONCLUSION

This study demonstrated that there were differences in alterations in structural and functional brain connectivity between patients with ESRD who were undergoing PD and HD and healthy participants. In addition, the language and verbal memory of patients who were undergoing HD were more impaired than those of patients who were undergoing PD. These findings suggest that brain connectivity and networks may be affected by different types of renal replacement therapy.

## CONFLICT OF INTEREST

None of the authors has any conflict of interest to disclose.

## AUTHOR CONTRIBUTION

Bong Soo Park and Myungjun Seong involved in writing‐original draft. Junghae Ko and Sihyung Park involved in methodology. Yang Wook Kim involved in supervision. Yoo Jin Lee, Seongho Park involved in review and editing. Il Hwan Kim and Jin Han Park involved in formal analysis. Kang Min Park involved in conceptualization.

## Data Availability

Research data are not shared.
